# DNA repair deficiency in neuropathogenesis: when all roads lead to mitochondria

**DOI:** 10.1186/s40035-019-0156-x

**Published:** 2019-05-09

**Authors:** Luis Bermúdez-Guzmán, Alejandro Leal

**Affiliations:** 1 Section of Genetics and Biotechnology, School of Biology, Universidad de Costa Rica, San José, 11501 Costa Rica; 2 Neuroscience Research Center, Universidad de Costa Rica, San José, Costa Rica

**Keywords:** DNA repair, Mitochondrial dysfunction, Neurodegeneration, Ataxia, PNKP

## Abstract

Mutations in DNA repair enzymes can cause two neurological clinical manifestations: a developmental impairment and a degenerative disease. Polynucleotide kinase 3′-phosphatase (PNKP) is an enzyme that is actively involved in DNA repair in both single and double strand break repair systems. Mutations in this protein or others in the same pathway are responsible for a complex group of diseases with a broad clinical spectrum. Besides, mitochondrial dysfunction also has been consolidated as a hallmark of brain degeneration. Here we provide evidence that supports a shared role between mitochondrial dysfunction and DNA repair defects in the pathogenesis of the nervous system. As models, we analyze PNKP-related disorders, focusing on Charcot-Marie-Tooth disease and ataxia. A better understanding of the molecular dynamics of this relationship could provide improved diagnosis and treatment for neurological diseases.

## Background

The present review aims to expand knowledge in the pathological process that involves defects in DNA repair and its interaction with mitochondrial dysfunction in neurodegeneration. Recently, evidence has grown on the involvement of mitochondrial dysfunction in neurodegenerative diseases of the central nervous system, such as Alzheimer’s and Parkinson’s and peripheral nervous system as well. Our team has worked mainly on the molecular causes of peripheral neuropathies that are classified as Charcot-Marie-Tooth disease (CMT). We identified a group of patients with mutations in the PNKP gene, an essential nuclear and mitochondrial DNA repair enzyme. Mutations in this gene have been associated with a pathological spectrum, varying from a neurodevelopmental impairment to a neurodegenerative disease. In this review, we analyze the existing literature that supports a pathological interaction between DNA repair and mitochondria, that causes an exclusive neurological effect, as in the case of PNKP-associated diseases.

## Main text

Nuclear and mitochondrial DNA are susceptible to damage due to errors of intrinsic DNA metabolism, and its exposure to radiation, reactive oxygen species and other environmental factors. Therefore, the DNA damage response is critical for cell survival and health [1].

**Fig. 1:**
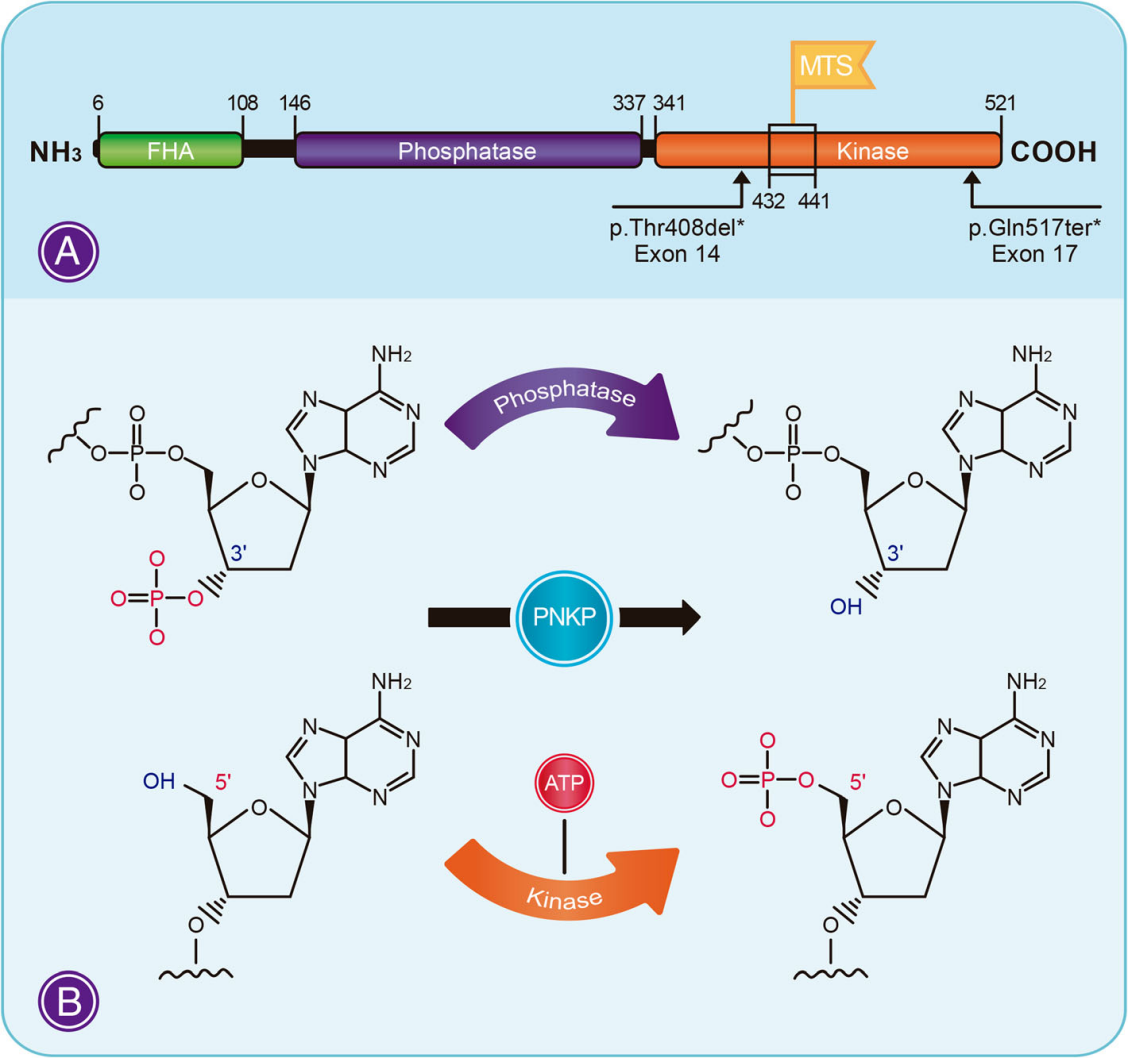
**a** Schematic representation of the functional domains of PNKP. We reported five unrelated patients with CMT2 disease, who were compound heterozygotes for the p.Thr408del (exon 14) and p.Gln517ter * (exon 17) variants [2]. Homozygous individuals for the p.Thr408del variant present Ataxia-ocular motor apraxia 4 (AOA4) [3]. **b** Enzymatic role of PNKP. The enzyme catalyzes the 3′-phosphate termini dephosphorylation and the phosphorylation of 5′-OH terminal by using ATP as the phosphate donor. Mitochondrial-targeting signal (MTS) is a necessary region for PNKP translocation to mitochondria. FHA: Fork-head-associated domain

The polynucleotide kinase 3′-phosphatase (PNKP) is the main enzyme responsible for restoring the 5′-phosphate and 3′-hydroxyl ends in DNA strand breaks, necessary for ligation during repair, especially in single strand breaks (SSBs) (Fig. 1) [4–6]. PNKP is recruited to repair errors in SSBs through interactions between its N-terminal FHA domain and the X-Ray Repair Cross Complementing one (XRCC1) protein, necessary for the recruitment of PNKP and other proteins [7]. In the case of double-strand breaks (DSBs), PNKP participates, in the non-homologous end joining pathway (NHEJ), which takes place when the FHA domain interacts with the X-Ray Repair Cross Complementing 4 protein (XRCC4) [8]. However, it was also demonstrated that DSBs end-joining reaction could be dependent on PARP1, PNKP (hPNK) and the XRCC1/LIG III complex, known as alternative NHEJ [9].

### SSB repair function in the nervous system: the model of PNKP in health and disease

Due to its high rate of oxygen consumption, the nervous system is susceptible to DNA damage, particularly in comparison with other tissues that contain non-replicating cell types. The brain metabolizes ~ 20% of consumed oxygen but has a low capacity to neutralize reactive oxygen species (ROS). Therefore, neurons are particularly susceptible to oxidative stress [10]. SSBs occur when one strand of the DNA losses a single nucleotide. If not repaired, it will represent a severe threat to genetic stability and cell survival. SSBs are considered the most common damage to DNA (1000 breaks/cell/day) in the cell [11], mainly derived from reactive oxygen species [12] and commonly repaired by the PNKP + XRCC1 complex. Since neurons have low levels of antioxidant enzymes and high transcriptional activity, these cells depend on the single-strand break repair pathway (SSBR) [13].

**Fig. 2:**
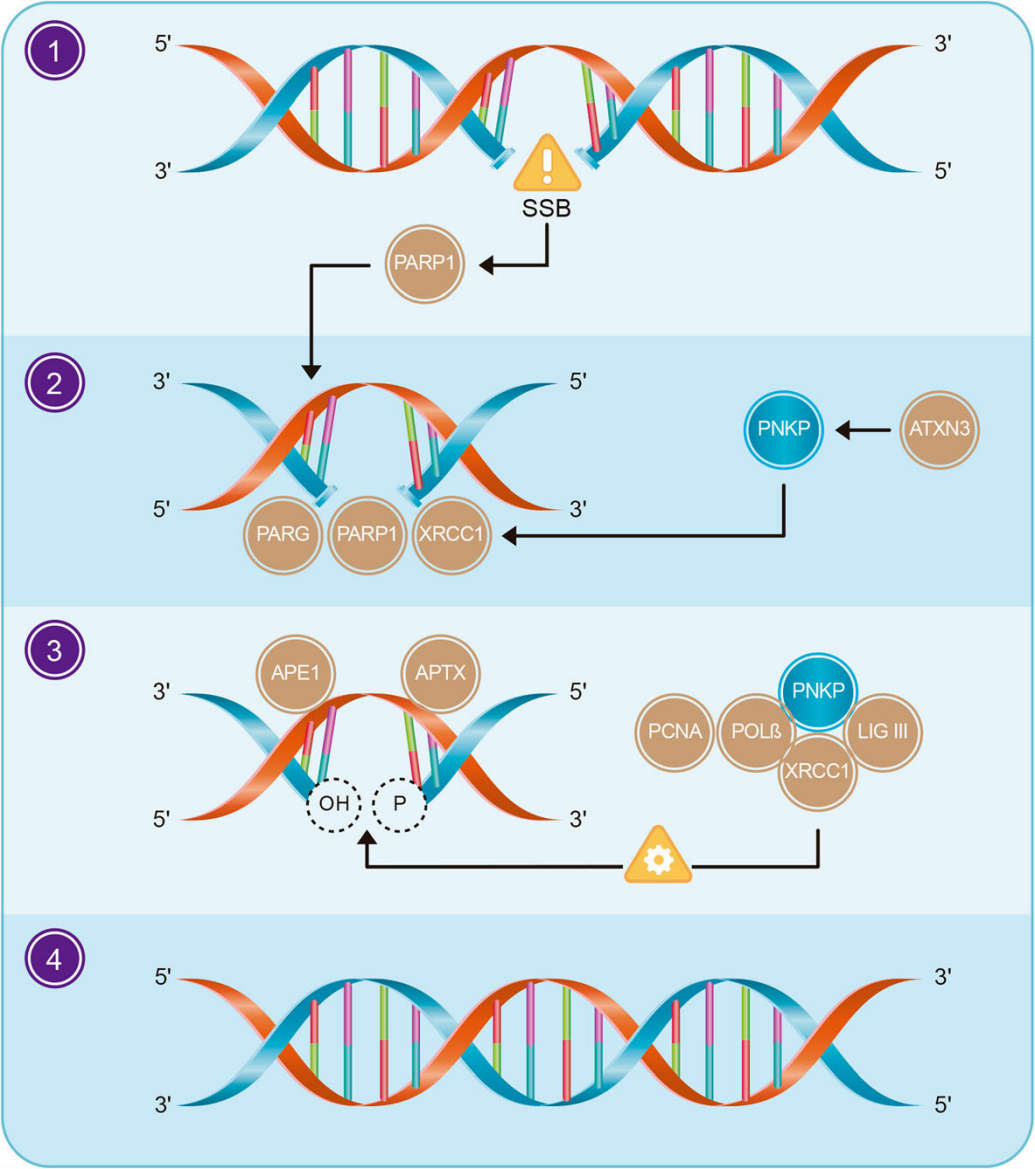
Basic stages of Single-Strand Break Repair: **1**. Detection of the break, **2**. DNA end processing, **3**. DNA gap filling and DNA ligation, **4**. Repaired strand. Direct breaks are detected by PARP1, which promotes the accumulation of different repair factors. Damaged termini are repaired by APE1, DNA polymerase (Pol β), PNKP and APTX. Ligase (LIG III) is needed to repair short-patch sites through a phosphodiester bond

SSBR proceeds in four stages: break detection, processing of DNA termini, DNA gap filling and ligation (Fig. 2). In the mature nervous system, genome stability related mechanisms differ from those operating during neurogenesis because of the lack of cell division and the absence of replicative DNA damage and homologous recombination repair [14]. Proliferating neural cells are proficient for DNA damage-induced apoptosis, [15] a resource to avoid excessive damage and control cell growth. In contrast, in post-mitotic cells, including neurons and cardiomyocytes, death by apoptosis is not that common and requires additional steps [16].

To complete the scenario, double-strand breaks (DSBs) can occur spontaneously in the brain, because of the high neuronal activity [17]. Nevertheless, in non-cycling cells, the homologous recombination system (HR) that repairs double-stranded breaks is unavailable [14]. Therefore, in post-mitotic neurons, NHEJ is the sole available pathway to repair this problem [18]. In the absence of DNA ligase IV, a key NHEJ component, neurons progressively accumulate endogenous DSBs [19]. Thus, in the mature nervous system, strand breaks and DNA modifications from oxidative damage can disrupt transcription and lead to cell death [20].

Despite the broad spectrum of diseases associated with mutations in DNA repair enzymes (such as immunodeficiency and higher cancer risk), defects in the SSBR pathway present a tendency to affect the nervous system, in some cases exclusively [21–23]. As PNKP has a vital role in both nuclear and mitochondrial DNA integrity, mutations in this enzyme or associated proteins, have several implications in the pathogenesis of neurological diseases. Until now, two different clinical manifestations have been associated with PNKP mutations: a neurodevelopmental impairment (Microcephaly and Seizures, MCSZ) and neurodegenerative disorders (i.e., Ataxia-ocular motor apraxia 4, AOA4) [24]. To illustrate the impact of DNA Repair Deficiency in neurological manifestations, we will explain some of the PNKP-associated diseases.

### SCA3

Spinocerebellar ataxia type 3 (SCA3) is an autosomal dominant neurodegenerative disease, and the most common inherited ataxia worldwide [25]. The disease is caused by an expansion of a polymorphic CAG trinucleotide repeat in the Ataxin 3 (ATXN3) C-terminal coding region. PNKP interacts with this protein and is inactivated by the mutant form of ATXN3, resulting in inefficient DNA repair [26]. This leads to an accumulation of DNA damage due to strand breaks with subsequent chronic activation of ATM signaling pathway.

### MCSZ

Some PNKP mutations cause an autosomal recessive disease characterized by microcephaly, seizures and developmental delay and behavioral problems (denoted MCSZ). In samples of patients severely affected with MCSZ [24] (family three: homozygous E326K mutation), and mildly affected individuals (family seven: compound heterozygous for T424GfsX48 in exon 14 and a 17-bp deletion in intron 15), concentrations of PNKP protein was much lower compared with unaffected family members. This low concentration was correlated with the severely affected individuals from families 1–6. Although all these mutations impair PNKP activity, family seven compound heterozygous individuals, present a slightly milder phenotype, probably due to a remnant function of the enzyme. Families four and five presented the same homozygous Thr424GlyfsX48 mutation.

In mice, a hypomorphic allele that reduces the expression of PNKP is sufficient for viability but affects genome integrity [27]. The complete loss of PNKP resulted in non-viable embryos. Therefore, endogenous DNA lesions cannot be properly repaired, leading to neural cell loss and resultant neuropathology. In their experiment, Shimada and colleagues found that PNKP inactivation resulted in the loss of cerebellar neuronal population in an ATM-independent, but partially p53-dependent manner [27]. Thus, they suggested that in MCSZ, the errors in neurons associated with high oxygen consumption, depend on PNKP. According to this, it was proposed that like in other syndromes, the microcephaly in MCSZ probably implicates defective double-strand break repair (DSBR) [28]. According to this, the instability of the encoding variants of PNKP rather than specific mutations causes both microcephaly and neurodegeneration. Thus, the lack of PNKP activity would be responsible for the mutagenic effects in neurons.

The role of PNKP and DSBs in the etiology of MCSZ can be supported by the fact that the process of DSBR needs XRCC4 instead of XRCC1 (involved in SSBs). As the E236K mutation modifies the phosphatase domain, the protein is defective in being recruited to DNA damage sites, possibly through decreased interaction with XRCC4-LigIV complex after DNA damage [29]. One explanation could be that low PNKP-E326K mutant levels in MCSZ do not result from mRNA instability but cellular protein instability. However, although this specific mutation has reduced DNA kinase and DNA phosphatase functions at 37 °C, normal activity was reported at 30 °C [5]. In MCSZ, E326K mutation represents an impaired electrostatic interaction between this region and XRCC4-Lig4 due to an amino acid charge swap [30]. Interestingly, among the seven families studied with MCSZ [24], the three families that shared a homozygous mutation that resulted in the E326K change, had the most severe phenotype, suggesting a major depletion in the protein functioning.

### AOA4

AOA4 is a rare autosomal neurodegenerative disorder characterized by ataxia, oculomotor apraxia, and peripheral neuropathy [3] caused by mutations in PNKP [28]. Muscle weakness progresses until most individuals start using wheelchairs by the second or third decade [30]. Interestingly, the fact that none of the mutations in MCSZ or AOA4 eliminates the phosphatase activity of the enzyme can be an indicator of lethal effect derived from a complete loss of its function [28]. This can be explained by the importance of the phosphatase activity in neurogenesis, since defects related to the 3′-phosphate end lack an effective alternative backup pathway. This lack was recently demonstrated in cancer cells lines [31]. Most of the mutations in PNKP that cause AOA4 belong to the kinase domain. Despite this, it is complicated to establish a phenotype-genotype correlation due to the compound heterozygous’ clinical picture. For example, Thr424GlyfsX48 mutation is present not only in MCSZ but in AOA4 [3, 24]. In this line, another case was also reported, in which two brothers were homozygous for the p.Thr424GlyfsX48 variant in PNKP [32]. They presented a severe progressive polyneuropathy and cerebellar atrophy, microcephaly, mild epilepsy, and intellectual disability. The authors were not able to establish a definitive diagnosis since these patients presented all clinical manifestations reported for PNKP mutations. Interestingly, mice homozygous for the p.T424GfsX48 allele were lethal at the embryonal stage [27]. Compound heterozygosity involving the p.Thr408del variant is also common in AOA4 and CMT2B2 patients [2, 3]. Recently, another research reported the case of a woman affected by AOA4 that also develop a pilocytic astrocytoma in the right cerebellar hemisphere [33]. The patient was compound heterozygous for p.T424GfsX49 / p.Tyr515ter. From the families affected by MCSZ [24], affected members of family seven were compound heterozygotes, and had the most favorable phenotype, carrying a non-coding mutation, which makes it possible to retain some PNKP activity. According to this, the phenotype generated by PNKP mutations is subject to allelic combination effects. Similar to AOA4 other neurological diseases are caused by mutations in genes that belong to the SSBR pathway. Ataxia with oculomotor apraxia-1 (AOA1) is caused by mutations in aprataxin (APTX), spinocerebellar ataxia with axonal neuropathy-1 (SCAN1) is derived from mutated Tyrosyl-DNA Phosphodiesterase 1 (TDP1). Since diseases such as AOA and SCAN1 tend to present a later onset in life, a possible explanation is that some backup mechanisms protect the cells during development [20]. These cells can even trigger apoptosis to avoid major damage in the nervous system. During neural development, progenitor cells undergo symmetric divisions that expand the size of the progenitor pool. After that, they will switch to an “asymmetric” mode of division, wherein each round produces one progenitor cell and one “postmitotic” neuron [13]. Differentiated cells can succumb to non-repaired DNA damage, but in this postmitotic scenario, apoptosis is likely to be avoided since neuronal cells are unreplaceable. Thus, as a consequence of the damage produced to this pool of cells, neurodegenerative diseases are likely to be related with DNA damage that cannot be repaired, or a progressive addition of the continuous damage of the DNA [14].

### Epileptic encephalopathies

A group of epileptic encephalopathies has also been associated with PNKP [34, 35]. PNKP contains a C-terminal mitochondrial-targeting signal (MTS) (Fig. 1). This signal is needed for the entrance of PNKP to the mitochondria, through the mitofilin protein. This MTS site, close to the carboxy-terminus of the protein, locates at codons 432–441 (ARYVQCARAA). In silico analysis indicated that mutations in this region would result in a lower affinity of the MTS “domain” because the protein failed to enter the mitochondria. Interestingly, the loss of the MTS resulted in a clear decrease in the PNKP’s activity during mtDNA repair. Seizures always represent an excessive acute energy demand, irrespective of their origin in the brain [36]. The relationship between mitochondria dysfunction and seizure generation could be deduced from changes in calcium homeostasis, oxidation of ion channels and neurotransmitter transporters by ROS. Secondary mitochondrial dysfunction is a well-known mechanism in some epileptic disorders, even for those diseases that are mainly of non-mitochondrial origin [37].

Many mutations in codons that involved the MTS “domain” of PNKP are related to infantile epileptic encephalopathy and other undefined conditions (PNKP[gene] - ClinVar - NCBI, 2018), and could be hypothesize that the etiology of the disease is linked to the inability of PNKP to translocate to neuronal mitochondria, or its failure to repair errors generated in the mtDNA. It has also been proposed that mitochondrial transport of Ca^2+^ has a role in the intracellular sequestration of this ion in neurons [38]. Due to their importance in the modulation of neuronal excitability and synaptic transmission, changes in Ca^2+^ homeostasis produced by errors in DNA repair (SSB), could be a direct explanation for the effects of mitochondrial dysfunction in the generation of seizures [36].

### Mitochondrial common etiology in axonal degeneration: using CMT as a model

Charcot-Marie-Tooth disease (CMT) is a heterogeneous hereditary motor and sensory polyneuropathy, which is the most common hereditary neuromuscular disorder. All patterns of inheritance have been related to CMT, but autosomal-dominant inheritance is the most common. Clinically, there are demyelinating (CMT1, CMT4) axonal (CMT2) and intermediate forms of the disease [39].

**Fig. 3:**
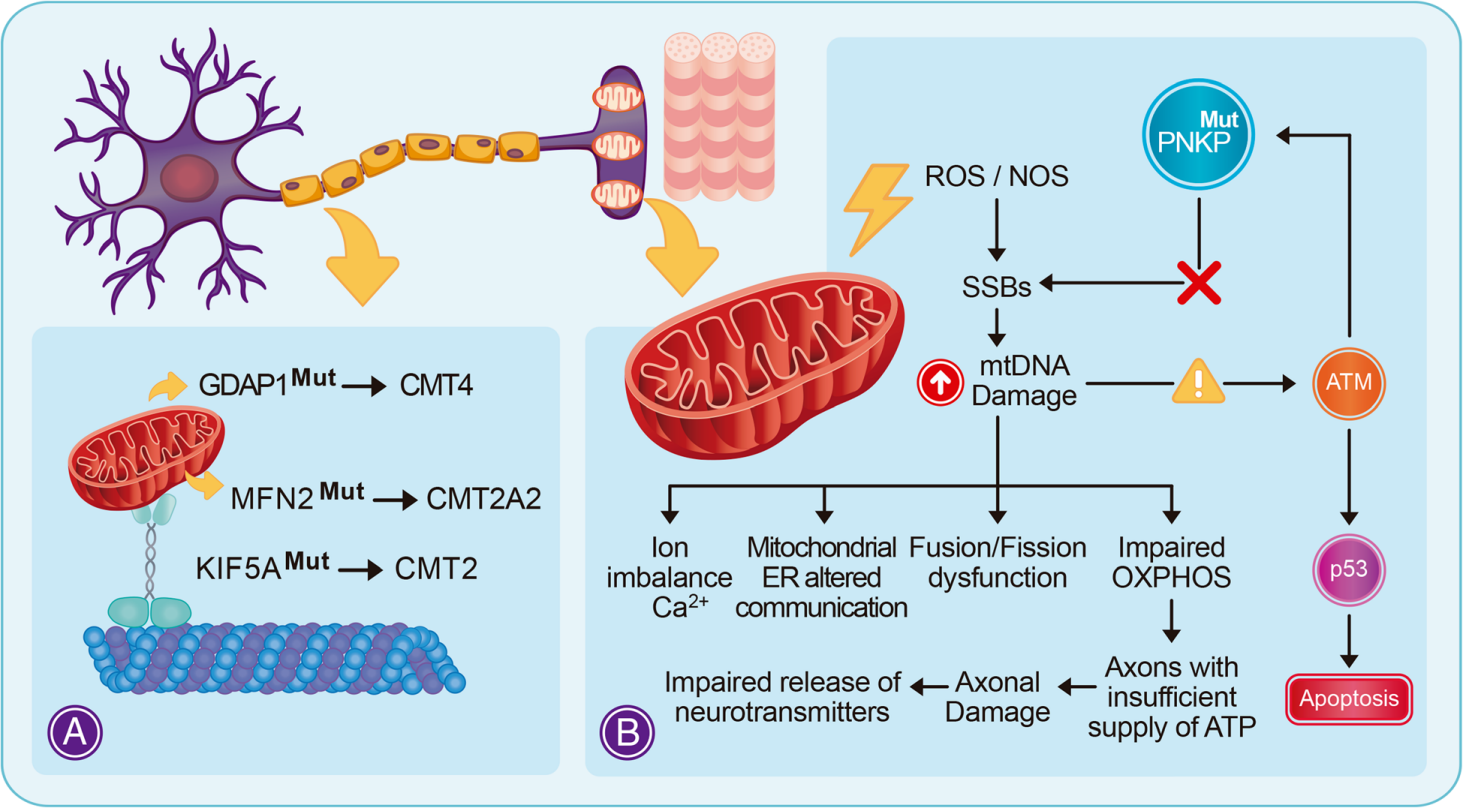
Integrative representation showing the mitochondrial dysfunction as an etiology of the axonal degeneration. **a** Mutations associated with mitochondrial dynamics can cause axonal damage in CMT2 phenotype. **b** Role of PNKP in the maintenance of mtDNA stability. When PNKP is mutated, ROS/NOS generate an environment of genotoxic stress, that can impair the normal axonal function. Since the glycolytic capacity of neurons is restricted, mitochondrial oxidative phosphorylation is essential for neuronal ATP supply [36]. ATM-dependent phosphorylation of PNKP at serines 114 and 126 in response to oxidative DNA damage inhibits degradation of PNKP. This PNKP stability is required for DNA repair [45]. ATM can also activate p53 which undergoes apoptosis in the presence of high levels of mtDNA damage. ER: endoplasmic reticulum. OXPHOS: oxidative phosphorylation

Peripheral nerves need an efficient, energetic metabolism to maintain their machinery related to the transport through axons, which may be as long as 1 m. Proper mitochondrial functioning is a critical factor for axonal and myelin formation and maintenance [39]. In fact, areas with high demands for ATP like synaptic terminals, active growth cones or axonal branches, contain more mitochondria than other cellular domains [40]. Defects in mitochondrial transport may cause local energy depletion and toxic changes in Ca^2+^ buffering that may trigger synaptic dysfunction [41]. The damage in the mitochondria transport system causes a similar pathology as those derived from primary mitochondrial impaired function. Both mechanisms lead to metabolic deficiencies, oxidative damage, cytotoxicity, and apoptosis [42]. This can affect the neuromuscular junction and lead to various forms of neuropathy, neurodegeneration, muscular dystrophy and paraplegia. In mature axons of cultured hippocampal neurons, an average transport velocity of approximately 0.5 μm/s was reported [43]. Assuming the same for motor axons, mitochondrion would reach its synaptic target in approximately 23 days [42]. Thus, the combination of distance and volume, seen from the intracellular transport system, could be the “Achilles heel” of neurons and axons [44]. In this context, we emphasize that mitochondrial transportation impairment is crucial in the process of axonal damage in CMT2 (Fig. 3a). Axonal peripheral neuropathy could also develop a secondary demyelinating phenotype. Even in that case, it is well known that proper mitochondrial functioning is a critical factor for myelin formation and maintenance [39]. The following genes have been involved in mitochondria-related axonal degeneration in CMT:

#### KIF5A

Kinesin Heavy Chain (KIF5A) mutations can cause axon degeneration limited to the peripheral nervous system [46]. This protein is implicated in the transport of mitochondria from the neural soma to the axon terminal. The first relationship between CMT and KIF5A was reported by [47]. These mutations were also found in Spastic paraplegia type 10 (SPG10). A single KIF5A mutation was also detected in a diagnosed CMT2 patient belonging to an SPG10 family [48]. In zebrafish, KIF5Aa mutants (heterozygous), exhibit a similar phenotype to CMT2, including the axonal degeneration. In addition, dominant homozygous mutant showed a peripheral sensory axons mitochondria deficiency and degeneration [49].

#### GDAP1

The Ganglioside-induced differentiation-associated protein 1 (GDAP1) is a mitochondrial fission factor mutated in CMT4 [50]. Mutations can cause perturbed axonal transport, and this may lead to impaired energy production. This process is also known in other neurodegenerative diseases due to mutations in genes related to mitochondrial dynamics [51]. Patients with recessive mutations usually have a severe axonal neuropathy with onset in early childhood. In contrast, patients with dominant GDAP1 mutations usually have a mild axonal neuropathy with later onset and slower disease progression [52]. A reduced intracellular antioxidant glutathione (GHS) concentration and reduced mitochondrial membrane potential were found in patients with GDPA1 mutations [53]. Therefore, an implication of oxidative stress in the pathogenesis of the disease is suggested.

Mutations in GDAP1 can lead to mild, persistent oxidative stress in the peripheral nervous system. However, the high oxidative stress can be compensated in the unaffected central nervous system by GDAP1L1 [54]. This protein acts in response to elevated levels of oxidized glutathione, by translocating from the cytosol to mitochondria, an essential process to substitute the loss of GDAP1 expression.

#### MFN2

Mitofusin 2 (MFN2) is a protein from the outer mitochondria membrane that participates in mitochondrial fusion. MFN2 is also necessary for axonal mitochondrial transport [55]. In a later work, the authors suggested that although the role of MFN2 mutations in CMT2 pathogenesis is not clear, axonal degeneration could be explained due to the abnormal transport of mitochondria through the microtubule system [56]. Thus, that mechanism could explain the peripheral neuropathy and possibly the pyramidal tract involvement, where the longest axons are the first and the main ones to be affected. It is interesting that both the mutations in GDAP1 associated with mitochondrial fission and MFN2, associated with mitochondrial fusion; generate a mainly axonal CMT phenotype. The underlying cause could be deregulation in the mitochondrial dynamics, either due to the inability to be transported through the axons or due to an in-situ dysfunction caused by the disruption of mitochondrial homeostasis. This allows us to propose the hypothesis of the involvement of PNKP in the regulation of the integrity of mtDNA and the pathogenesis of CMT2. An idea that we will develop later.

There is enough evidence about pathogenic mutations that disrupt the mitochondrial homeostasis in the scenario of axonal damage. A particular case of mutations that affect mtDNA integrity is the group of mutations in the mitochondrial specific DNA polymerase (POLG). These mutations can cause an autosomal recessive ataxia-neuropathy syndrome, with onset in the first three decades of life [57]. This ataxia, named mitochondrial-associated ataxia syndrome (MIRAS), is caused by multiple mtDNA deletions. Symptoms include peripheral neuropathy, myoclonus, and epileptic seizures. The replication defects derived from mutations in POLG affect components of the mtDNA-encoded electron transport chain and oxidative phosphorylation [14].

The dysfunction of the mitochondrial network also has a critical role in diseases like chemotherapy-induced peripheral neuropathies (CIPN). The effects of chemotherapeutic drugs on mitochondria in the peripheral nervous system have three principal axes of impairment: calcium signaling, reactive oxygen species (ROS) and apoptosis [58]. In an experimented with the *Wld*^S^ mutant mouse that expresses a chimeric nuclear protein Nmnatl, a protective role of this protein was shown due to an inhibition of axonal degeneration. This axonal damage was mainly caused during rotenone and vincristine treatment [59]. This suggests that this protein protects axons by reducing ROS accumulation or toxicity. Neurons treated with vincristine in the dorsal root ganglia (DRG) decreases the rate of mitochondrial dynamics, including fusion, fission, and motility; and this led to mitochondrial fragmentation and visible axon degeneration [60]. Interestingly, overexpression of an agent called cytNmnat1 inhibited the axon degeneration by preserving the normal mitochondrial dynamics. If this approach can be tested in patients affected with axonal degeneration, like CMT2B2, a possible future therapeutic target is in sight.

### PNKP mutations could lead to disease through mitochondrial dysfunction

As previously mentioned, functionally active full-length PNKP is present in mitochondria as well as nuclei [69]. The idea about the contribution of mitochondria dysfunction in neurological disease is not new, not even in CMT. The contributions of the principal mutations in CMT affecting mitochondrial homeostasis have been extensively reviewed [39, 61], so the role of mitochondria in distal axonal degeneration in different peripheral neuropathies [62].

The local energy demand is known to be different between neuronal compartments, and it tends to change over time. The peripheral terminals of sensory axons represent sites of an exceptionally high ATP demand during conduction [40]. Axons consume high amounts of ATP, especially to fuel the sodium/potassium ATPase or sodium pump that functions to remove the sodium ions that enter the axon during impulse activity [63]. Because of their energy demand, most neuronal mitochondria reside in axons and dendrites [64]. As neurons cannot switch to glycolysis when oxidative phosphorylation (OXPHOS) becomes limited, mitochondrial transport, together with the dynamic processes of mitochondrial fission and fusion, facilitates the transmission of energy across long distances [65]. Whereas mitochondria play a crucial role in axonal function, it has been proposed that mtDNA is more susceptible to oxidative damage because of its lacks of protective histones [66]. Another fact is that in contrast to nDNA, almost all mtDNA is transcribed. Therefore, unrepaired mutations in the mtDNA could alter energy production and are expected to influence cell homeostasis. Thus, mtDNA genomic stability is critical for neuronal functioning [67]. The location of the mitochondria at the nerve terminals is the possible responsible for the increased susceptibility damage [68]. This vulnerable situation can be associated with age-related dysfunction and oxidative damage when compared with non-synaptic mitochondria.

In this regard, mitochondrial PNKP may play a critical role: PNKP depletion was observed to cause a significant decrease in mtDNA repair. The full restoration of repair of the H_2_O_2_-induced strand breaks in mitochondria required both the kinase and phosphatase activities of PNKP [69] (Fig. 3b). The authors concluded that PNKP plays a major role in the repairing of oxidized bases in the mitochondrial genome. Indeed, the major source of mtDNA lesions derives from the vast quantities of ROS produced in the mitochondria during respiration [68]. If this damage is not repaired, the mitochondria will not be able to function properly, and this can lead to neuropathy.

In the mitochondria, the base excision repair (BER) and SBBR pathways are the primary DNA repair mechanisms [70]. In this line, another work demonstrated that PNKP-depleted human mitochondrial extracts showed a significant decrease in both BER and SSBR activities. In fact, PNKP was found to be the major 3-phosphatase within mitochondria [71]. Various mtDNA lesions, including point mutations and large-scale deletions of mtDNA, can lead to mitochondrial dysfunction and cellular apoptosis [20]. In fact, mtDNA oxidative stress induces strand breaks with a much higher frequency than mutagenic lesions [72]. These lesions represent an impairment for both replication and transcription of mtDNA. When the damage rises above a threshold level, it can induce degradation of mtDNA molecules [73]. Future clinical research should also analyze the mtDNA damage in DNA repair deficiency, as it could be useful as a biomarker in patients with mutations in PNKP and other enzymes from the SSBR pathway.

### Mitochondrial dysfunction as a common signature in cerebellar degeneration

Cerebellar degeneration is a common neurological feature in ataxia. An interesting fact about the cerebellum is its extensive development after birth. During this phase, replication stress is predictable due to the rapid cell proliferation, leading to more DNA breaks than in other parts of the nervous system [67]. The cerebellar degeneration that leads to the ataxia presenting in patients with mitochondrial dysfunction can be primary (caused by affecting the cerebellum and its connections), or secondary (due to loss of proprioception derived from peripheral neuropathy or neuronopathy, or a combination of both) like in spinocerebellar ataxia [74].

Cerebellar development starts with the neurogenesis of Purkinje cells (PC) around embryonic day E10.5. Their proliferation ceases shortly before birth, but in the postnatal period, they still grow, mature and develop their synaptic network [75]. Purkinje neurons possess vast numbers of synaptic connections via their dendritic tree, which implies a high-energy demand for the integration of excitatory and inhibitory synaptic inputs and subsequent maintenance of ion gradients. One abnormality in PC mitochondria is observed in the pcd mouse, a recessive model of neurodegeneration, involving cerebellum and retina. It is derived from mutations in the Agtbp1 gene (*Nna1*), which expresses a protein that translocates to mitochondria. Agtbp1 loss of function results in altered bioenergetics and mitochondrial dysfunction. As a result, mice present progressive gait ataxia [76]. Following this idea, we summarize several genes that are involved in this pathogenic mechanism based on mitochondrial-related cerebellar degeneration.

#### Sacs

Autosomal recessive spastic ataxia of Charlevoix-Saguenay (ARSACS) is a childhood-onset neurological disease characterized by cerebellar ataxia with spasticity, pyramidal syndrome and peripheral neuropathy. It is caused by mutations in the *SACS* gene encoding sacsin. The knockout mice, display age-dependent neurodegeneration of cerebellar PC due to impaired mitochondrial network and the accumulation of mitochondria in the soma and proximal dendrites [77].

#### LIG iii

The inactivation of Lig3 in the mouse nervous system results in mtDNA loss leading to profound mitochondrial dysfunction, disruption of cellular homeostasis and incapacitating ataxia [78]. Morphological abnormalities of the mitochondria at postnatal times coincided with the emergence of ataxia. This was demonstrated in cerebellar PC neurons that showed distorted mitochondrial cristae structure and broad changes in its morphology. There was also a marked reduction of complex III and IV immunostaining.

#### APTX

Although cytoplasmic aprataxin may be present at a low level in all cell types, it seems to be more predominant in neuron and neuron-like cell lines and tissues [73]. The expression of the total aprataxin transcript and aprataxin MTS-encoding transcript varies among the different brain regions but were more abundant in the cerebellum. Aprataxin knockdown had a significantly higher level of ROS than control cells, lower citrate synthase activity, and reduced mtDNA copy number [73]. As the protein has different isoforms (differing from each other at the N- and C- termini), not every isoform has the MTS sequence.

#### TDP1

TDP1 hydrolyzes the phosphodiester bond at a DNA 3′-end linked to a tyrosyl moiety and is involved in the repair of topoisomerase I (Top1) DNA covalent complexes [79]. A fraction of the TDP1 encoded by the nuclear gene translocates to mitochondria [82]. As mitochondrial base excision repair depends on TDP1 activity, it is required for efficient repair of oxidative damage in that organelle [80–82].

Cerebellar neurons and primary astrocytes derived from Tdp1−/− mice present an inability to rapidly repair DNA SSBs associated with Top1–DNA complexes or oxidative damage [83]. Moreover, loss of the protein resulted in age-dependent and progressive cerebellar atrophy in spinocerebellar ataxia with axonal neuropathy-1 (SCAN1) patients. The overexpression of a toxic form of mitochondrial topoisomerase I (TOP1mt*) generates excessive mitochondrial protein-linked DNA breaks (mtPDBs), that results in a TDP1-dependent compensatory upregulation of mitochondrial gene transcription. Misassembled of the ETC complex III resulted in the absence of TDP1 due to the imbalance in transcription of mitochondrial- and nuclear-encoded electron transport chain (ETC) subunits. Bioenergetics profiling further reveals that TDP1 promotes oxidative phosphorylation under both basal and high-energy demands [84].

#### ATXN1

In cerebellar tissue of a Purkinje cell-driven spinocerebellar ataxia type 1 (SCA1), mice display deficits in cerebellar OXPHOS complex I (NADH-coenzyme Q oxidoreductase) [85]. In SCA1, Complex I genes are upregulated at the time of onset and upregulation persists into the late-stage disease.

SCA1 transgenic mice present clinical features of cerebellar dysfunction. The loss of PC is evident in both heterozygous and homozygous six-month-old mice. However, apoptosis does not seem to be involved in PC degeneration. While levels of brain-derived mtDNA are not different between SCA1 and control mice, mtDNA levels are significantly reduced in cerebellum of SCA1 mice [86].

#### ATXN3

In SCA3, the interaction of PNKP with mutant ATXN3 markedly abrogate PNKP’s enzymatic activity and DNA repair efficacy, resulting in persistent accumulation of strand breaks. Mutant ATXN3 potently activates the DNA damage-response ATM signaling pathway [26]. In SCA3, complex II exhibits a consistent tendency toward decreased activity in the presence of expanded ataxin-3, particularly in differentiated PC [87]. In neuronal cultures of the cerebellum, striatum and substantia nigra, the polyglutamine-expanded ataxin-3 (Q79) activates mitochondrial apoptotic pathway and leads to neuronal death by upregulating Bax expression and downregulating Bcl-xL expression of cerebellar, striatal or substantia nigra neurons [88].

The presence of the full-length mutant ataxin-3 in Q71 homozygotes is either positive in cerebellum and over the cerebral cortex. However, the fragment and aggregate of ATXN3 were detected more abundantly in the cerebellum [89]. Truncated mutant ATXN3 led to more mitochondrial fission due to the lower expression of Mfn-1 and Mfn-2. In transgenic mouse models, truncated mutant ATXN3 also led to mitochondrial dysfunction, neurodegeneration and cell death in the cerebellum [90].

#### POLG

In POLG-related neurodegeneration, the primary consequence of POLG mutation in neurons is mtDNA depletion. Progressive accumulation of mtDNA deletions and point mutations was found accompanied by increasing numbers of complex I–deficient neurons [91]. Severe Purkinje and granule cell loss and Bergmann gliosis in some patients characterized these lesions.

#### FXN

Friedreich’s ataxia is an autosomal recessive inherited neurodegenerative disorder, caused by an expansion mutation within intron 1 of the FXN gene. It is characterized by progressive spinocerebellar ataxia, cardiomyopathy, scoliosis, and diabetes. The frataxin-deficient YG8R mouse model showed a limitation of the maintenance of mitochondrial membrane potential in cerebellar granule neurons. Specifically, a deficiency in Complex I increased levels of mitochondrial and cytosolic ROS. Excessive ROS production results in oxidative stress and diminishes the level of glutathione (GSH) [92]. Deficient expression of frataxin leads to deleterious alterations in iron metabolism in the mitochondrial matrix that is presumed to play a crucial role in the oxidative damage and subsequent degenerative features of this disease [93].

Despite the known role of mitochondria dysfunction in cerebellar degeneration that usually leads to ataxia, it is not clear why diseases such as POLG and Friedreich’s ataxia occur with many systemic clinical manifestations. Interestingly, ataxia associated with mitochondrial dysfunction seems to be the primary clinical manifestation, if caused by mutations in the enzymes of the SSBR pathway.

#### ATM

**Fig. 4:**
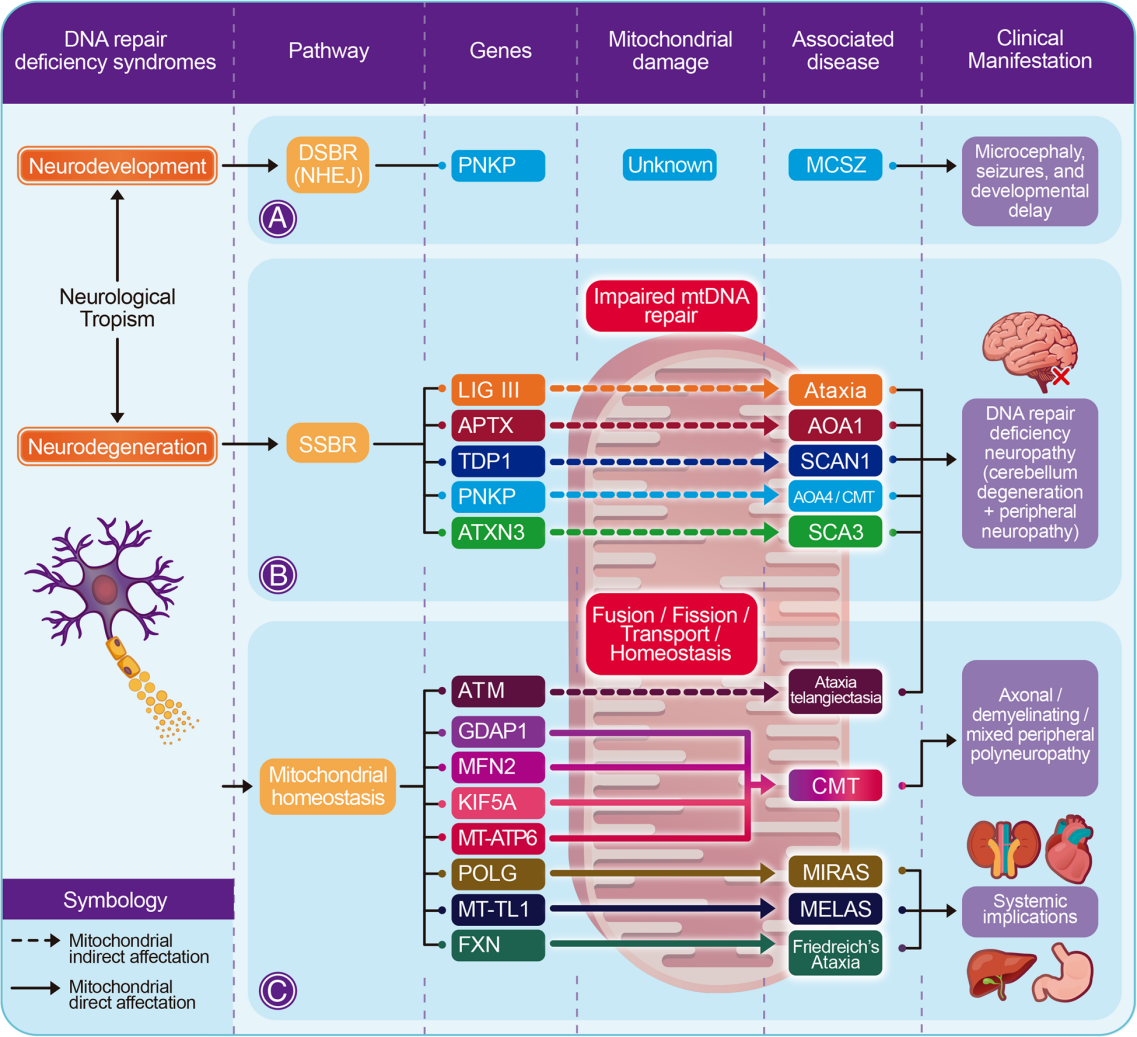
Genotype and phenotype correlation according to the type of mitochondrial damage. DSBR is involved in neurodevelopmental impairment without mitochondrial involvement evidence (**a**), whereas neurodegenerative disorders proceed from SSBR deficiency or mitochondrial homeostasis dysfunction. In SSBR related diseases, the protein variants cause an indirect effect on mitochondria that lead to exclusively neurological impact characterized mainly by peripheral neuropathy and cerebellum atrophy (**b**). On the other hand, mutations associated with mitochondrial functioning itself have direct systemic implications (**c**). Although both clinical categories have mitochondrial involvement, DNA repair deficiency is the primary cause of neuropathological tropism (discussed later). MIRAS: mitochondrial recessive ataxia syndrome / MELAS: mitochondrial myopathy encephalopathy lactic acidosis and stroke

Ataxia-telangiectasia (AT) has been the archetypal neurological disorder associated with DNA double-strand break repair, mainly because of its predisposition to cancer [94]. The disease has an extended phenotype, as a cause of the dual function of ATM: DNA repair and regulation of oxidative stress and mitochondrial homeostasis. Although AT presents defects in DNA repair and mitochondrial dysfunction, the clinical manifestations have a systemic pattern. Thus, the pathological role of DNA repair deficiency in AT seems to extend to all cells in the body, which explains the predisposition to cancer, something rarely seen in other types of ataxia. In contrast, the mitochondrial dysfunction seems to be more crucial in neurons, especially in the cerebellum as recently demonstrated [95–97] (Fig. 4).

In some other the diseases caused by mutations in DNA repair enzymes, there was no clarity regarding the possible mitochondrial involvement. Recently, in Xeroderma pigmentosum A (XPA), a disease characterized by dermatological ailments but also progressive cerebral and cerebellar atrophy, sensorineural hearing loss and neuropathy, mitochondrial dysfunction was shown. The dysfunction seems to originate from defects in mitophagy, possibly mediated by hyperactivation of PARP1 [98–100]. Another interesting case is a recent report of a patient with mutations in XRCC1 who presented motor ocular apraxia, axonal neuropathy, and progressive cerebellar ataxia [101]. In this case, the authors also determined that hyperactivation of PARP1 was the cause of cerebellar ataxia. To understand the implication of this pathway in the mitochondrial dysfunction, it is also important to examine the PARP1/NAD^+^/SIRT1 axis. When PARP1 is activated, it orchestrates DNA repair, which leads to the loss of NAD^+^ and acetyl-CoA. This results in inhibition of SIRT1 that leads to an increase in mitochondrial ROS, owing to decreased activation of stress response factors such as AMPK, FOXOs, and PGC1α. Also, SIRT1 has been shown to regulate several DNA repair pathways positively; including xeroderma pigmentosum group A-complementing protein (XPA), KU70, ataxia telangiectasia mutated (ATM) and thymine DNA glycosylase (TDG) [102]. In this regard, PARP1 appears as a common potential therapeutic target for mitochondrial dysfunction in DNA repair disorders.

### Principles of DNA repair deficiency disorders

DNA repair deficiencies (DRD) result from different mutations in DNA repair enzymes. There are critical considerations to understand the molecular basis of several neurological diseases, and therefore, for establishing the correlation between the genotype and phenotype. From the available data, here we summarize the interactions of a group of elements that combined, can explain the molecular pathogenesis of these diseases.

The first consideration that should be taken into account is whether a *pathological tropism* (PT) is observed in the disease. PT can be understood as the tendency of a disease to affect a particular tissue. In the case of the SSBR pathway, it would mean an exclusive neurological effect [21]. This is possibly explained by the vulnerabilities of the nervous system regarding higher oxidative damage and higher dependence on DNA repair, compared to the rest of the body.

DRD neurological disorders can be divided into those affecting the neurons during development and the ones that affect the mature nervous system. In the process of neurogenesis, the replication stress associated with high rates of proliferation is a major source of DNA damage that can perturb neural development. Indeed, it can be the main etiology involved in neurodevelopmental disease. On the other hand, in the mature nervous system (post-mitotic cells) the high transcriptional activity associated with metabolism result in cell dysfunction and possibly death.

The next consideration to comprehend the specific pathology related to a mutation is the *momentum*, which is the stage of cellular development when the damage occurs. In the case of neurons, it can be mitotic or post-mitotic. Neurodevelopment-associated damage is more likely to originate in the mitotic momentum, and neurodegenerative diseases would begin principally post-mitotic. This is relevant to understand and classify the pathologies of the central nervous system. We suggest a more in-depth evaluation of the categories established for DRD disorders, especially those that appear to arise in the development but has neurodegenerative implications. Evidence indicates that AOA causative mutations affect the development of Purkinje cells through the impairment of mitochondrial homeostasis. Nevertheless, clinical manifestations occur in the first decade and progress through life, showing a neurodegenerative pathology. *Momentum* matters, since the most common types of DNA damage in the nervous system, are different at both stages, just like the repair systems available. In developing cells, the most important source of errors is the DNA replication itself. As we explained before, in post-mitotic neurons, the main source of mutations is the stress associated with transcription and the high-energy demand, in which the mitochondria are involved, primarily due to the ROS production [68]. This is what we define as the *source of mutagenic damage*.

The damage derived from mitotic cells is predominately double-strand breaks (DSBs), normally repaired by homologous recombination and the NHEJ (PNKP+XRCC4 complex). If breaks cannot be repaired, it would be lethal at this stage through apoptosis. In post-mitotic neurons, non-homologous end joining (NHEJ) is the sole pathway available to prevent accumulation of DNA double-strand breaks [103]. On the other hand, SSBs represent a serious threat to genetic stability and cell survival, especially because neurons have low levels of antioxidant enzymes and high metabolic activity. Proliferative cells become increasingly dependent on SSBR, and post-mitotic cells depend on SSBR exclusively, in which PNKP has a crucial role, especially in mitochondria-derived oxidative stress damage [11, 13]. In mature cells, the high frequency of error linked to SSBs and the lack of alternative pathways, especially in the mitochondria, could lead to an accumulation of damage. This may explain the late onset and slow progression of neurodegenerative diseases. Therefore, depending on the *momentum* and *source*, it is possible to classify the specific type of error in DNA: this can be defined as the principle of the (single or double) *affected strand*.

As observed for PNKP, XRCC1 is required in the neurogenesis of cerebellar interneuron [104]. XRCC1 deficiency drives a progressive and persistent accumulation of strand breaks in mature neuronal populations. According to this, it is possible to notice an overlap between the phenotype caused by mutant variants from the same pathway (SSBR), especially in the impaired development of the cerebellum. It could be hypothesized that the clinical spectrum can also be related to the level of disruption in the SSBR cascade.

Since PNKP could share the same mutations in both neurodevelopmental and neurodegenerative diseases, the problem cannot be referred to the protein variant itself, but how the PNKP mutant interacts with the available repair machinery for a specific *momentum* and *strand*. This can be synthesized as the effect of the mutation in the enzyme and the particular pathway involved that ultimately leads to a specific phenotype. In tissues like the cerebellum where mutations arise with a very high rate (due to the high proliferation rate), and axons, where the energy demand is so high that it compromises mitochondrial homeostasis, mutations in DNA repair enzymes could lead to the observed neurodegenerative phenotype. Thus, at this point, it is possible to integrate the mitochondrial dysfunction in both axonal and cerebellar degeneration and introduce the last principle or consideration: *mitochondrial intervention*.

Since the energetic requirement of neurons is very high, axons contain more mitochondria than other cellular domains. This mitochondrial activity generates a high amount of ROS with the subsequent SSBs. The cerebellum cells, such as PC, have a high rate of postnatal replication, which is highly dependent on mitochondrial activity. Most diseases with ataxia as primary clinical manifestation due to mutations in DNA repair enzymes (SSBR) present an indirect effect of mitochondrial function (Fig. 4b). Therefore, although the primary damage is more likely to derive from unrepaired nuclear DNA damage (associated with replication stress), there is a concomitant role between nuclear and mitochondrial genome dysfunction as the underlying pathogenic process. According to this, we propose a model in which mutations in DNA repair enzymes such as PNKP, APTX, TDP1, ATXN1/3, and others, cause an indirect effect on mitochondria that lead to exclusively neurological impact due to the specific metabolic and genome maintenance characteristics of the central nervous system. This damage is mainly peripheral neuropathy and cerebellum atrophy, showing an evident mitochondrial SSBR-dependent dysfunction (Fig. 4b). To determine the moment in which the mitochondrial damage occurs could be important for a future therapeutic approach.

Diseases such as Friedreich’s ataxia and MIRAS (caused by POLG mutations) present an extended phenotype, maybe derived from the equal vulnerability that these variants confer to the mitochondria in the whole organism. Even though the clinical difference is evident, still more research is needed to understand the molecular basis of this systemic damage compared with the diseases that tend to present a pathological tropism. One idea is that mtDNA often shows an uneven distribution between cells of the same tissue, so this can explain why defects appear only above a mutation-specific threshold in vulnerable organs. This can generate a mosaic pattern of mitochondrial dysfunction and differences in the distribution patterns of the pathogenic mutations can be observed and be clinically relevant [36].

## Conclusions

Deficiency in DNA repair can lead to multiorgan diseases or can be confined to the nervous system. Enzymes such as PNKP, TDP1, and APTX, are not only active in the nucleus of neurons but also in their mitochondria. They constitute an example of how the deficiency in the DNA SSBR machinery converges with mitochondrial dysfunction, in the etiology of pathologies with an exclusive neurological involvement. There is enough evidence to conclude that mitochondrial dysfunction is what best explains the clinical manifestations and progression in neurodegeneration. Therefore, intensive research should be directed to develop new therapeutic strategies, targeting the common feature of these diseases: the mitochondria.

## Abbreviations

AOA: Ataxia-oculomotor apraxia
APTX: Aprataxin
ATM: Ataxia-telangiectasia mutated
ATXN3: Ataxin 3
CIPN: Chemotherapy-induced peripheral neuropathies
CMT: Charcot-Marie-Tooth
DDR: DNA repair deficiency
DSBR: Double-strand break repair
DSBs: Double-strand breaks
FXN: Frataxin
GDAP1: Ganglioside-induced differentiation-associated protein 1
GHS: Glutathione
KIF5A: Kinesin Heavy Chain 5A
MCSZ: Microcephaly and Seizures
MELAS: Mitochondrial myopathy encephalopathy lactic acidosis and stroke
MFN2: Mitofusin 2
MIRAS: Mitochondrial-associated ataxia syndrome
mtDNA: Mitochondrial DNA
MTS: Mitochondrial-targeting signal
nDNA: Nuclear DNA
NHEJ: Non-homologous end joining
PC: Purkinje cell
PNKP: Polynucleotide kinase 3′-phosphatase
POLG: Polymerase γ
ROS: Reactive oxygen species
SCA3: Spinocerebellar ataxia type 3
SCAN1: Spinocerebellar ataxia with axonal neuropathy-1
SPG10: Spastic paraplegia type 10
SSBR: Single strand breaks repair
SSBs: Single strand breaks
TDP1: Tyrosyl-DNA Phosphodiesterase 1
XRCC1/4: X-Ray Repair Cross Complementing 1/4
